# Characterization of prehospital time delay in primary percutaneous coronary intervention for acute myocardial infarction: analysis of geographical infrastructure-dependent and -independent components

**DOI:** 10.1186/s12942-023-00328-5

**Published:** 2023-03-30

**Authors:** Keisuke Oyatani, Masayuki Koyama, Nobuaki Himuro, Tetsuji Miura, Hirofumi Ohnishi

**Affiliations:** 1 Department of Public Health, Sapporo Medical University School of Medicine, S-1, W-17, Chuo-Ku, Sapporo, 060-8556 Japan; 2 Department of Pediatrics, Sapporo Medical University School of Medicine, Sapporo, Japan; 3 Department of Cardiovascular, Renal and Metabolic Medicine, Sapporo Medical University School of Medicine, Sapporo, Japan; 4 Department of Clinical Pharmacology, Faculty of Pharmaceutical Sciences, Hokkaido University of Science, Sapporo, Japan

**Keywords:** Prehospital delay, Percutaneous coronary intervention (PCI), ST-elevation myocardial infarction (STEMI), Onset-to-door time (ODT), Geographical infrastructure, Minimum prehospital system time (min-PST), Estimated delay-in-arrival-to-door (eDAD), Health care systems

## Abstract

**Background:**

Prehospital delay in reaching a percutaneous coronary intervention (PCI) facility is a major problem preventing early coronary reperfusion in patients with ST-elevation myocardial infarction (STEMI). The aim of this study was to identify modifiable factors that contribute to the interval from symptom onset to arrival at a PCI-capable center with a focus on geographical infrastructure-dependent and -independent factors.

**Methods:**

We analyzed data from 603 STEMI patients who received primary PCI within 12 h of symptom onset in the Hokkaido Acute Coronary Care Survey. We defined onset-to-door time (ODT) as the interval from the onset of symptoms to arrival at the PCI facility and we defined door-to-balloon time (DBT) as the interval from arrival at the PCI facility to PCI. We analyzed the characteristics and factors of each time interval by type of transportation to PCI facilities. In addition, we used geographical information system software to calculate the minimum prehospital system time (min-PST), which represents the time required to reach a PCI facility based on geographical factors. We then subtracted min-PST from ODT to find the estimated delay-in-arrival-to-door (eDAD), which represents the time required to reach a PCI facility independent of geographical factors. We investigated the factors related to the prolongation of eDAD.

**Results:**

DBT (median [IQR]: 63 [44, 90] min) was shorter than ODT (median [IQR]: 104 [56, 204] min) regardless of the type of transportation. However, ODT was more than 120 min in 44% of the patients. The min-PST (median [IQR]: 3.7 [2.2, 12.0] min) varied widely among patients, with a maximum of 156 min. Prolongation of eDAD (median [IQR]: 89.1 [49, 180] min) was associated with older age, absence of a witness, onset at night, no emergency medical services (EMS) call, and transfer via a non-PCI facility. If eDAD was zero, ODT was projected to be less than 120 min in more than 90% of the patients.

**Conclusions:**

The contribution of geographical infrastructure-dependent time in prehospital delay was substantially smaller than that of geographical infrastructure-independent time. Intervention to shorten eDAD by focusing on factors such as older age, absence of a witness, onset at night, no EMS call, and transfer via a non-PCI facility appears to be an important strategy for reducing ODT in STEMI patients. Additionally, eDAD may be useful for evaluating the quality of STEMI patient transport in areas with different geographical conditions.

**Supplementary Information:**

The online version contains supplementary material available at 10.1186/s12942-023-00328-5.

## Background

Treatment of ST-elevation myocardial infarction (STEMI) requires rapid revascularization of the affected vessel to achieve the best clinical outcomes. Primary percutaneous coronary intervention (primary PCI) is the recommended treatment for STEMI patients [1–3], and early revascularization (within 2–4 h of symptom onset) is particularly effective [4, 5]. Door-to-balloon time (DBT), or the interval it takes to perform PCI once a patient arrives at a facility, is an emphasized measure of the success of STEMI treatment. Current guidelines recommend a DBT of 90 min or less [1–3]. However, DBT represents only a part of total ischemic time (TIT), the time interval from the onset of coronary occlusion to reperfusion. Shortening DBT further may not significantly reduce TIT, as shortening of DBT has already been achieved in most PCI facilities in developed countries [6]. The primary opportunity to reduce TIT in STEMI patients now lies in the prehospital time [7, 8].

A study in Denmark showed that first-medical-contact-to-balloon time (FMCTB), which accounts for the majority of TIT from prehospital to posthospital, was a significant predictor of mortality in STEMI patients treated with primary PCI [9]. DBT, the latter component of the FMCTB, is not expected to have an intervention effect, as discussed above. TIT can be divided into prehospital onset-to-door time (ODT) and posthospital DBT, with ODT depending on multiple factors such as the patient's decision time, distance from the STEMI onset site to the nearest PCI facility, and the mode of patient transportation. Modifiable components of ODT that can improve the clinical outcomes of STEMI patients have yet to be fully elucidated. In addition, the impact of geographical factors, which are also essential for a healthcare system, on variations in ODT has not been adequately considered in previous studies. The aim of this study was to identify modifiable elements of ODT for STEMI patients with consideration of geographical factors.

## Methods

### Hokkaido Acute Coronary Care Survey and data collection

Hokkaido is the largest prefecture in Japan with a population of 5,381,733 and an area of 83,424 km^2^, accounting for 22% of the total area of the country. Located at latitudes similar to those of major European cities, Hokkaido is home to 21 medical regions with varying sizes and distributions of medical resources, including 84 PCI facilities as of 2015. Of these facilities, 47 are located in three urban regions: Sapporo Medical Region, Kamikawa-Chubu Medical Region, and Minami-Oshima Medical Region (Additional file 1: Figure S1A, B) [10]. Local governmental fire defense headquarters in Japan provide emergency medical services (EMS), dispatch of the nearest available ambulance to the site of an emergency call and transport to the nearest hospital that can accept patients. Ambulances are funded by local governments through tax revenue and are free for patients to use. Regions with a population of less than 150,000 are allocated one ambulance per 30,000 people, while regions with a population greater than 150,000 are allocated three ambulances with an additional ambulance for each additional 60,000 people. Each ambulance is staffed by three crew members trained in rescue, transportation, and medical emergencies. However, it is not standard for the EMS team to present an electrocardiogram from the site to the destination.

We performed a retrospective analysis using anonymized data from the Hokkaido Acute Coronary Care Survey, which was conducted under the initiative of the Hokkaido Government in 2014–2016. This registry-based survey collected data on clinical characteristics, elapsed time, and outcomes of STEMI in patients for two months in the summers of 2014 and 2015 and for 2 months in the winters of 2015 and 2016. The study included 72 of the 84 PCI facilities in Hokkaido and consecutive STEMI patients within seven days of symptom onset were enrolled in the study. STEMI was defined according to the current guidelines [1–3], and patients with hospital-onset STEMI were not included in the registry. The inclusion criteria for the present analyses were STEMI presentation on admission and primary PCI conducted within 12 h of symptom onset as the primary treatment. Patients with missing data for the time course of transport and treatment and patients with STEMI complicated by out-of-hospital cardiac arrest (OHCA) were excluded from the analyses.

Data were collected by trained personnel at each participating hospital and included data for patient characteristics such as age, sex, Killip class and Japan Coma Scale (JCS) [11] on arrival at the PCI facility, OHCA, onset place (at a non-personally identifiable level), presence or absence of witnesses, history of prior acute myocardial infarction (AMI), and details on the time course of symptoms, transport, and treatment. The cerebral performance category (CPC) at discharge or 4 weeks after admission was recorded as an outcome.

The study was approved by the Ethics Committee of Sapporo Medical University School of Medicine (#3-1-18) and conducted in accordance with the Declaration of Helsinki. Informed consent was not obtained owing to the use of anonymized data in this observational study.

### Definitions of types of transport and time intervals

**Fig. 1:**
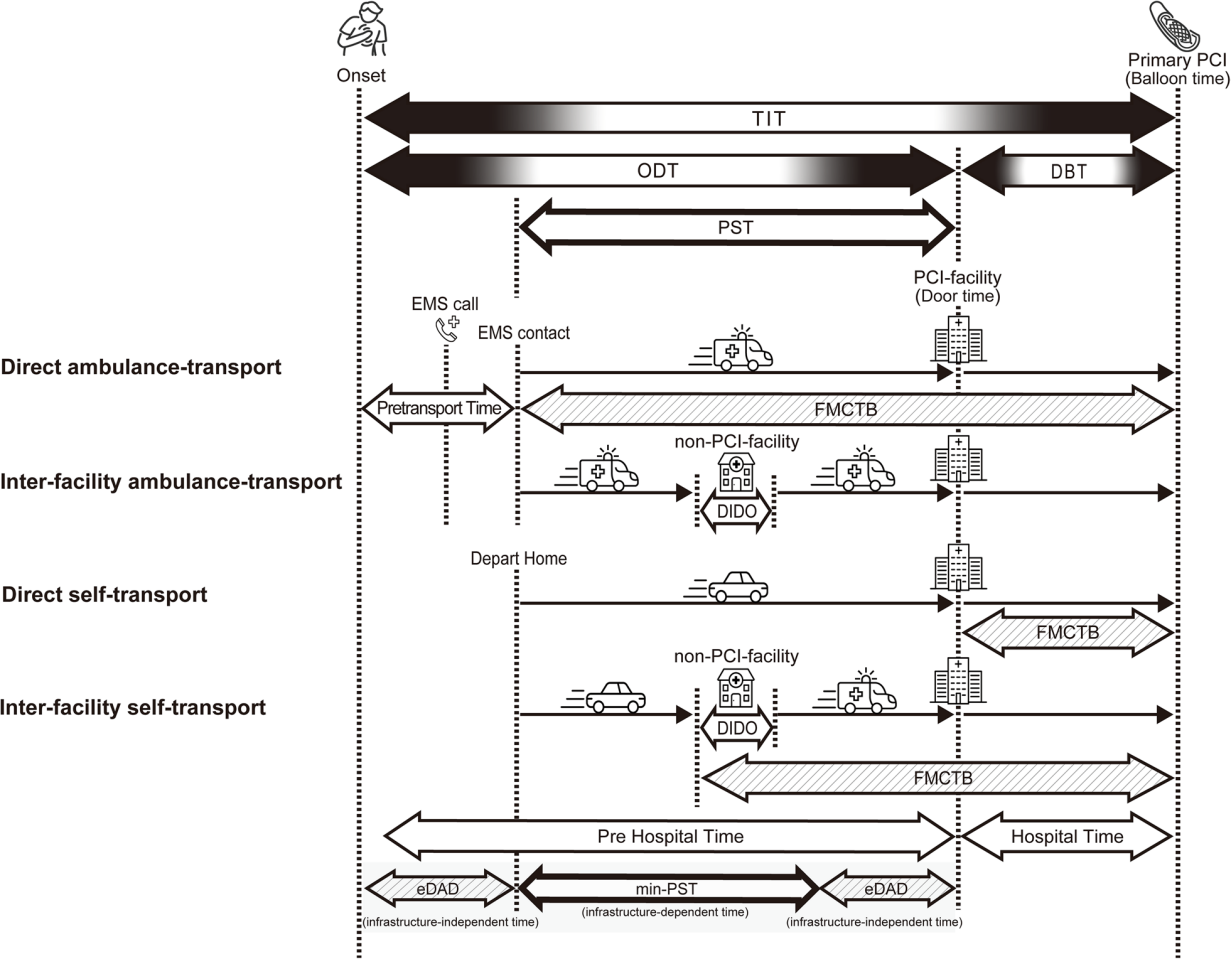
Pathways and time courses of transport to the primary PCI. In prehospital time, geographical infrastructure-independent time (eDAD) exists both immediately after the onset of illness and at other times. An example of the former is the time of hesitation before taking action to see a doctor, and an example of the latter is the time spent for transport via a non-PCI facility. *TIT* total ischemic time, *PST* prehospital system time, *ODT* onset-to-door time, *DBT* door-to-balloon time, *FMCTB* first-medical-contact-to-balloon time, *DIDO* door-in-to-door-out time. We simulated min-PST (travel time from each onset point to the nearest PCI facility) using ArcGIS Pro (ESRI, Inc., Charlotte, NC, USA). eDAD (estimated delay-in-arrival-to-door; ODT minus min-PST) means the amount of room in prehospital time for improvement

The patients were divided into two groups according to the type of transportation to PCI facilities: patients who were directly admitted to PCI facilities (Direct admission group) and patients who were transferred via a non-PCI facility to a PCI facility (Inter-facility transfer group). The groups were subdivided into four types of transport to a PCI facility as shown in Fig. 1: Direct ambulance-transport for patients who were directly admitted to PCI facilities by the EMS, Inter-facility ambulance-transport for patients who were transported via a non-PCI facility to a PCI facility by the EMS, Direct self-transport for patients who directly visited a PCI facility, and Inter-facility self-transport for patients who visited a non-PCI facility and were subsequently transported to a PCI facility by the EMS. Time intervals were defined as follows. TIT was defined as the interval from onset of symptoms to primary PCI, pretransport time was defined as the interval from onset of symptoms to EMS contact, ODT was defined as the interval from onset of symptoms to door time (DT), and DBT was defined as the interval from DT to balloon time (BT).

### Calculations of time required for transport to the nearest PCI facility and time delay in arrival at the PCI facility

Geographical information system (GIS) software is a tool that has previously been used to model accessibility to health care services [12, 13]. We incorporated data for the spatial location of STEMI onset and the locations of PCI facilities into ArcGIS Online and ArcGIS Pro (ESRI, Inc., Charlotte, NC, USA) to estimate the minimum prehospital system time (min-PST) for each patient, which is the minimum time required for ground transportation to a PCI facility along the road network. We also introduced a new measure called the estimated delay-in-arrival-to-door (eDAD), which is calculated by subtracting the min-PST from the ODT. In the prehospital period, geographical infrastructure-independent time (eDAD) exists both immediately after the onset of illness and at other times. An example of the former is the time of hesitation before taking action to see a doctor, and an example of the latter is the time spent on transport via a non-PCI facility. eDAD is the maximum time interval that can be modulated to shorten ODT after eliminating geographical infrastructural factors.

In this study, we tentatively defined the target ODT as ≤ 120 min, corresponding to a TIT ≤ 180 min if DBT is assumed to be 60 min. To calculate min-PST, we used comprehensive street data from ESRI as of 2021 with static travel times derived from historical average automobile speeds and routes following traffic rules. We did not consider variations in factors, such as the day of the week, time of day, or weather, that may affect travel time.

### Clinical outcomes

Because echocardiography data were unavailable in the survey, CPC in patients at discharge or four weeks after the onset of STEMI was used as a clinical endpoint. Following a previous report [14], we defined poor outcome as CPC 3, 4, or 5 (i.e., death or severe disability) and good outcome as CPC 1 or 2, with poor outcome as the objective variable in our analysis.

### Statistical analysis

Continuous numeric variables are expressed as mean ± standard deviation (SD) or medians with interquartile range (IQR). Categorical variables are presented as numbers and percentages (%). Categorical variables were compared using the χ^2^ test or Fisher's exact test when appropriate. Differences in continuous variables between groups were tested using the Student's t-test, Wilcoxon rank-sum test, or the Kruskal–Wallis test, depending on the distribution of variables. The normal distribution of variables was tested using the Kolmogorov–Smirnov test. A multiple comparison test following the Kruskal–Wallis test was performed using the Dann-Bonferroni method. Continuous variables were divided into clinically meaningful reference values, medians, or quartiles. Multivariate logistic regression analyses were performed to assess factors associated with prolonged eDAD, prolonged TIT and its time components, and poor outcomes. Factors used for constructing logistic regression models were that relevances are clear from previously [15–19] or those we selected from the clinical characteristics and transport time intervals. Separate models of clinical outcomes were used with consideration of collinearity. JMP^®^ Pro 16.2.0 (SAS Institute Inc., Cary, NC, USA) was used for the analysis. A two-sided P-value < 0.05 was considered statistically significant for all analyses.

## Results

### Clinical characteristics

**Fig. 2:**
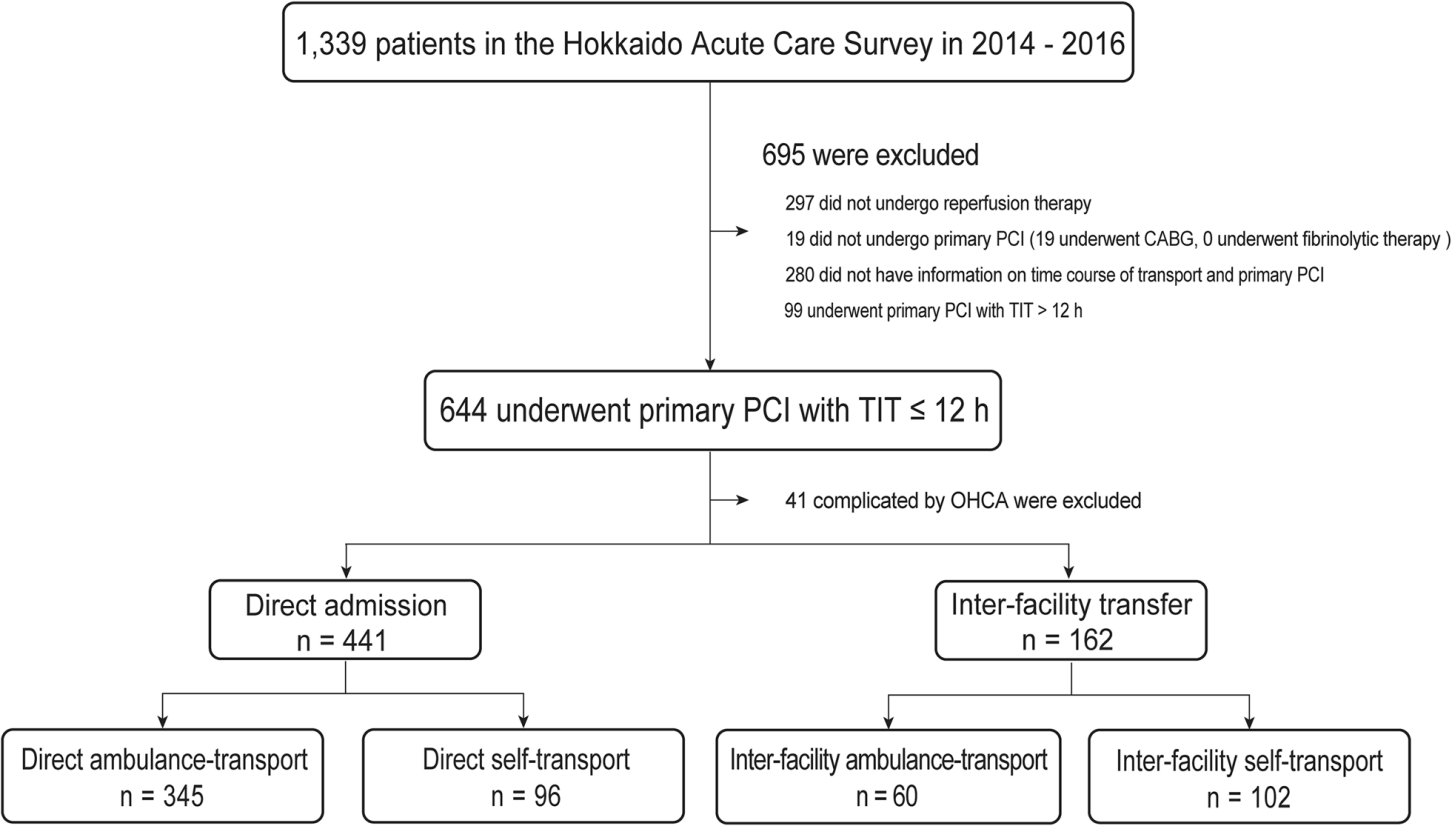
Flow chart for selection of the study population. *CABG* coronary artery bypass grafting, *TIT* total ischemic time, *OHCA* out-of-hospital cardiac arrest

As shown in Fig. 2, 1023 of the 1339 patients with STEMI registered in the Hokkaido Acute Coronary Care Survey underwent primary PCI, and none of the patients received fibrinolytic therapy. Based on the inclusion and exclusion criteria, 603 patients with STEMI were included in this analysis. The mean age of those patients was 68 years and 22.9% were females. Clinical characteristics of the study subjects and comparisons of subjects in the Direct admission group and those in the Inter-facility transfer group are shown in Table 1. The proportion of patients in urban medical regions and the proportion of patients who called EMS were larger in the Direct admission group than in the Inter-facility transfer group. On the other hand, the proportion of patients in rural medical regions and the proportion of patients who did not call the EMS were larger in the Inter-facility transfer group than in the Direct admission group. Furthermore, the Direct admission group had shorter TIT and ODT and longer DBT than those in the Inter-facility transfer group.

**Table 1:** Baseline and procedure characteristics

	All (n = 603)	Direct admission (n = 441)	Inter-facility transfer (n = 162)	P value
Demographic characteristics and history				
Age, years (mean [SD])	67.9 (12.5)	68.0 (12.3)	67.4 (13.1)	0.564
≥75 years, n (%)	192 (31.8)	140 (31.8)	52 (32.1)	0.934
Women, n (%)	138 (22.9)	95 (21.5)	43 (26.5)	0.195
Previous MI, n (%)	69 (11.4)	55 (12.5)	14 (8.6)	0.329
Presentation at PCI facility				
JCS on arrival				
0/I, n (%)	558 (92.5)	403 (91.4)	155 (95.7)	0.085
II/III, n (%)	30 (5.0)	27 (6.1)	3 (1.9)	
Unknown, n (%)	15 (2.5)	11 (2.5)	4 (2.5)	
Killip class on arrival				
1/2, n (%)	418 (69.3)	301 (68.3)	117 (72.2)	0.639
3/4, n (%)	88(14.6)	67 (15.2)	21 (13.0)	
Unknown, n (%)	97 (16.1)	73 (16.6)	24 (14.8)	
Comparison of situations at symptom onset				
Location at STEMI onset				
Urban medical regions, n (%)	311 (51.6)	265 (60.1)	46 (28.4)	<0.001*
Rural medical regions, n (%)	292 (48.4)	176 (39.9)	116 (71.6)	
Witness				
Yes, n (%)	311 (51.6)	237 (53.7)	74 (45.7)	0.079
No, n (%)	292 (48.4)	204 (46.3)	88 (54.3)	
Season				
Summer, n (%)	282 (46.8)	202 (45.8)	80 (49.4)	0.462
Winter, n (%)	321 (53.2)	239 (54.2)	82 (50.6)	
Day of onset				
Weekday, n (%)	416 (69.2)	307 (69.9)	109 (67.3)	0.533
Weekend or holiday, n (%)	185 (30.8)	132 (30.1)	53 (32.7)	
Time of onset				
Daytime, n (%)	365 (60.5)	261 (59.2)	104 (64.2)	0.264
Nighttime, n (%)	238 (39.5)	180 (40.8)	58 (35.8)	
Transport path				
EMS call				
Yes, n (%)	405 (67.2)	345 (78.2)	60 (37.0)	<0.001*
No, n (%)	198 (32.8)	96 (21.8)	102 (63.0)	
Use of air transportation, n (%)	7 (1.2)	0	7 (4.2)	–
Mechanical support/interventions additional to primary PCI				
IABP, n (%)	71 (11.8)	53 (12.0)	18 (11.1)	0.887
CABG,n (%)	10 (1.7)	7 (1.6)	3 (1.9)	0.733
Time course				
TIT, min (median [IQR])	185 (125, 290)	160 (114, 255)	259 (170, 359)	<0.001*
ODT, min (median [IQR])	104 (56, 204)	80 (50, 153)	197 (120, 305)	<0.001*
ODT ≤ 120 min achieved, n (%)	339 (56.2)	298 (67.6)	41 (25.3)	<0.001*
DBT, min (median [IQR])	63 (44, 90)	67 (47, 95)	54 (35, 70)	<0.001*
DBT ≤ 90 min achieved, n (%)	454 (75.3)	318 (72.1)	136 (84.0)	0.003*
FMCTB, min (median [IQR])	98 (71, 144)	88 (66, 200)	130 (95, 193)	<0.001*
FMCTB ≤ 120 min achieved, n (%)	405 (67.2)	332 (75.3)	73 (45.1)	<0.001*
DIDO, min (median [IQR])	40 (13, 72)	–	40 (13, 72)	
Outcome				
Death (CPC 5), n (%)	38 (6.3)	26 (5.9)	12 (7.4)	0.498
Poor (CPC 3-5), n (%)	55 (9.1)	38 (8.6)	17 (10.5)	0.478
Good (CPC 1-2), n (%)	548 (90.9)	403 (91.4)	145 (89.5)	

We compared categorical variables using the χ^2^ test or Fisher's exact test and continuous variables using Student's t-test or the Wilcoxon rank-sum test. Variables are expressed as numbers (%), means (SD: standard deviation), or medians (IQR; interquartile ranges). MI, myocardial infarction; JCS, Japan Coma Scale; urban medical regions, Sapporo, Kamikawa Chubu, and Minami Oshima medical regions; rural medical regions, regions outside the urban medical regions; Daytime, 6:00 a.m–6:00 p.m; Nighttime, 6:00 p.m–6:00 a.m; EMS, emergency medical services; PCI, percutaneous coronary intervention, IABP, intra-aortic balloon pump therapy; CABG, coronary artery bypass grafting; TIT, total ischemic time; ODT, onset-to-door time; DBT, door-to-balloon time; FMCTB, first-medical-contact-to-balloon time; DIDO, door-in-to-door-out time; CPC, cerebral performance category

*P < 0.05

### TIT and its time components

**Fig. 3:**
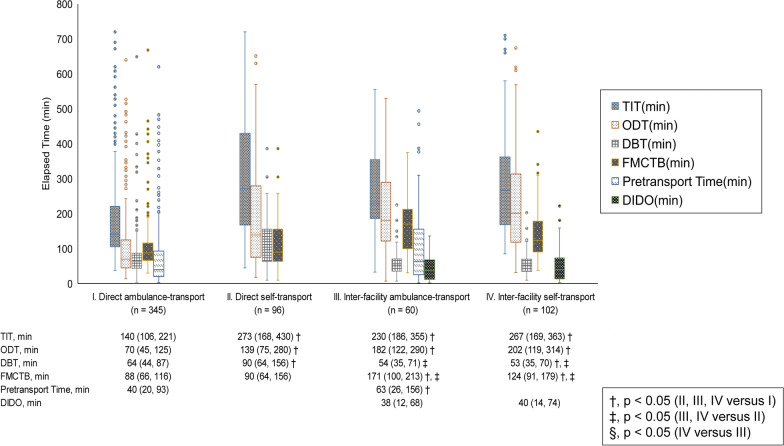
Comparison of time delays to reperfusion with different transport pathways. Data are expressed as medians (interquartile range). P values for the overall comparisons were calculated using the Wilcoxon rank-sum test or the Kruskal–Wallis test with the Dann-Bonferroni method. ^†^p < 0.05 (II, III, IV versus I); ^‡^p < 0.05 (III, IV versus II); ^§^p < 0.05 (IV versus III). *TIT* total ischemic time, *ODT* onset-to-door time, *DBT* door-to-balloon time, *FMCTB* first-medical-contact-to-balloon time, *DIDO* door-in-to-door-out time

TIT and data for its time components in the four types of transportation to PCI facilities are shown in Fig. 3. The Direct ambulance-transport group had the shortest TIT and ODT among the four groups. ODT accounted for the majority of TIT in all groups. DBT in the Direct self-transport group was longer than that in the Inter-facility self-transport group, but there was no significant difference between the two groups in TIT. The median door-in-to-door-out time was 38 min (IQR 12–68 min) in the Inter-facility ambulance-transport group and 40 min (IQR 14–74 min) in the Inter-facility self-transport group (p = 0.57).

Pretransport time was shorter in the Direct ambulance-transport group than in the Inter-facility ambulance-transport group. The results of multiple logistic regression analysis for factors associated with pretransport time > 43 min (median pretransport time) are shown in Table 2. Age ≥ 75 years and onset at night were independently associated with pretransport time > 43 min, while onset in winter was an independent negative factor for prolonged pretransport time. A rural medical region was not associated with pretransport time prolongation. The time from onset to the EMS call, a component of pretransport time, was significantly shorter in winter, though the time from the EMS call to the first medical contact was not significantly different between winter and summer (Additional file 1: Table S1).

**Table 2:** Factors associated with prolongation of pretransport time, ODT, and TIT

	Pretransport time > 43 min			ODT > 104 min			TIT > 185 min		
	OR	95% CI	P value	OR	95% CI	P value	OR	95% CI	P value
Age ≤ 64 years	1	[Ref.]		1	[Ref.]		1	[Ref.]	
65–74 years	1.46	0.87–2.44	0.15	1.21	0.76–1.91	0.42	1.76	1.13–2.74	0.01*
≥ 75 years	1.88	1.14–3.10	0.01*	1.86	1.19–2.91	< 0.01*	2.37	1.54–3.65	< 0.01*
Women	0.74	0.45–1.23	0.25	0.72	0.45–1.13	0.16	0.85	0.55–1.31	0.45
Recurrent MI	1.21	0.65–2.26	0.55	0.96	0.54–1.71	0.90	1.22	0.70–2.12	0.48
No witness	1.46	0.97–2.20	0.07	1.69	1.17–2.44	< 0.01*	1.52	1.07–2.17	0.02*
Onset in winter	0.62	0.41–0.94	0.03*	0.64	0.44–0.93	0.02*	0.88	0.61–1.26	0.47
Onset on weekends/holiday	1.02	0.66–1.58	0.93	1.03	0.69–1.54	0.89	1.14	0.77–1.67	0.52
Onset at night	1.70	1.12–2.57	0.01*	1.36	0.93–1.98	0.11	1.44	1.00–2.08	< 0.05*
Killip class 3/4 on arrival	0.88	0.49–1.57	0.67	1.14	0.66–1.94	0.64	1.52	0.90–2.55	0.11
No EMS call				2.92	1.91–4.49	< 0.01*	3.22	2.12–4.87	< 0.01*
Medical region and transport way									
Urban (direct admission)	1	[Ref.]		1	[Ref.]		1	[Ref.]	
Rural (direct admission)	1.15	0.72–1.82	0.56	1.17	0.77–1.77	0.47	1.14	0.75–1.72	0.54
Urban (inter-facility transfer)	2.94	0.66–13.02	0.16	2.73	1.27–5.85	< 0.01*	1.67	0.80–3.48	0.17
Rural (inter-facility transfer)	1.74	0.91–3.33	0.09	8.04	4.41–14.68	< 0.01*	3.58	2.12–6.05	< 0.01*

Adjusted odds ratio (OR) and 95% confidence interval (CI) from logistic regression analysis indicating the likelihood of pretransport time > 43 min (median pretransport time) in Ambulance Transfer groups, ODT (onset-to-door time) > 104 min (median ODT), and TIT (total ischemic time) > 185 min (median TIT). OR > 1 indicates increased odds of prolongation of each time component. Respective reference categories = age ≤ 64 years, male gender, first occurrence of MI, presence of a witness, onset in summer, onset in the daytime, Killip class 1/2 on arrival, EMS call, and urban medical region (direct admission). CPC, cerebral performance category; MI, myocardial infarction; Daytime, 6:00 a.m–6:00 p.m; Nighttime, 6:00 p.m–6:00 a.m; urban medical regions, Sapporo, Kamikawa Chubu, and Minami Oshima medical regions; rural medical regions, regions outside the urban medical regions

* P < 0.05

Factors associated with ODT > 104 min (median ODT) in the logistic regression analysis are shown in Table 2. Age ≥ 75 years, no witness, no EMS call, and inter-facility transfer were independent positive factors for prolonged ODT. Onset in winter was an independent negative factor for prolonged pretransport time.

Factors associated with TIT > 185 min (median TIT) in the logistic regression analysis are shown in Table 2. Age of 65–74 years, ≥ 75 years, no witness, onset at night, no EMS call, and onset in a rural medical region with inter-facility transfer were independent positive factors for prolonged TIT.

### Relationships between eDAD, min-PST, and ODT

eDAD (i.e., ODT minus mPST) could be calculated in 594 cases with detailed geographical data for the onset location. The median min-PST was very short (3.7 min), but min-PST data varied widely on a patient-to-patient basis, with the maximum min-PST being 156 min (Additional file 1: Figure S2). The characteristics of patients divided by median eDAD (83.5 min) are shown in Additional file 1: Table S2. The percentages of patients in the short eDAD group who had onset in urban medical regions, who did not across medical regions (i.e., were treated at a PCI facility in the same medical area as the onset location), and who called the EMS were higher than the percentages of patients in the long eDAD group. There was no significant difference in DBT between the two groups.

**Fig. 4:**
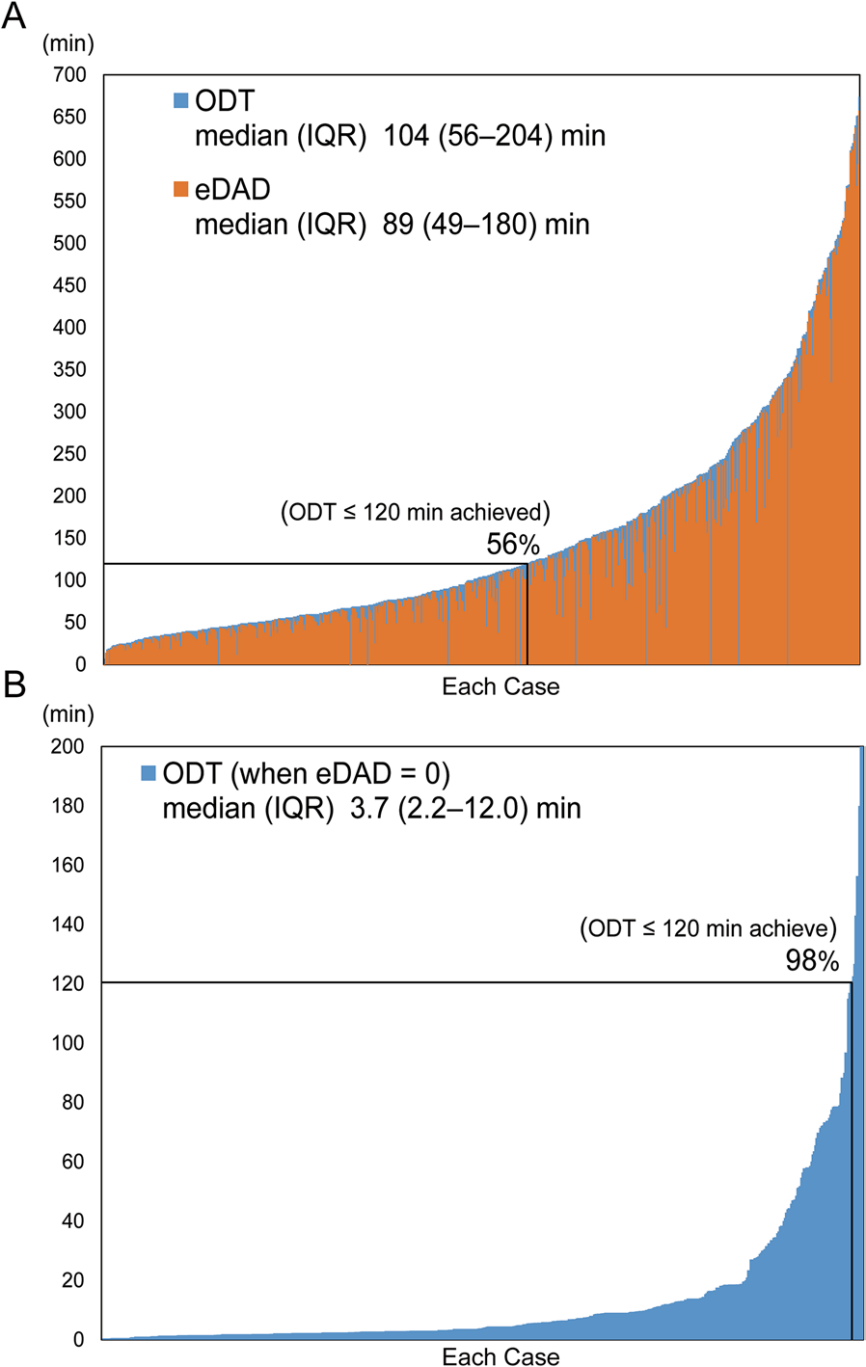
Cumulative frequency distribution of ODT. **A** Cumulative frequency distribution of ODT and eDAD for each case. **B** Projected cumulative frequency distribution of ODT when eDAD = 0 is postulated. The percentage of cases with ODT ≤ 120 min is shown in each panel. *ODT* onset-to-door time, *eDAD* estimated delay-in-arrival-to-door

Multiple logistic regression analysis for eDAD > 89.1 min (median eDAD) showed that age ≥ 75 years, no witness, onset at night, no EMS call, and inter-facility transfer in urban or rural medical regions were independently associated with prolonged eDAD (Table 3). Figure 4A shows histograms of ODT and eDAD for each case: 44% of the cases had ODT > 120 min. However, when eDAD was postulated to be zero in all patients, the percentage of patients with ODT ≤ 120 min was projected to be 98% (Fig. 4B).

**Table 3:** Factors associated with prolongation of eDAD

	eDAD > 89.1 min		
	OR	95% CI	P value
Age ≤ 64 years	1.00	[Ref.]	
65–74 years	1.25	0.80–1.97	0.33
≥ 75 years	2.01	1.29–3.12	< 0.01*
Women	0.70	0.45–1.09	0.12
Recurrent MI	0.96	0.54–1.69	0.89
No witness	1.64	1.14–2.35	0.01*
Onset in winter	0.82	0.57–1.18	0.29
Onset on weekends/holiday	1.03	0.69–1.53	0.89
Onset at night	1.68	1.16–2.44	< 0.01*
Killip class 3/4 on arrival	0.94	0.55–1.59	0.81
No EMS call	3.17	2.08–4.83	< 0.01*
Medical region and transport way			
Urban (direct admission)	1.00	[Ref.]	
Rural (direct admission)	0.98	0.65–1.50	0.94
Urban (inter-facility transfer)	3.16	1.40–7.11	< 0.01*
Rural (inter-facility transfer)	3.57	2.11–6.07	< 0.01*

Adjusted odds ratio (OR) and 95% confidence interval (CI) from logistic regression analysis indicating the likelihood of eDAD (estimated delay-in-arrival-at-the-door) > 89.1 min (median eDAD). OR > 1 indicates increased odds of prolongation of each time component. Respective reference categories = age ≤ 64 years, male gender, first occurrence of MI, presence of a witness, onset in summer, onset in the daytime, Killip class 1/2 on arrival, EMS call, and urban medical region (direct admission). CPC, cerebral performance category; MI, myocardial infarction; Daytime, 6:00 a.m–6:00 p.m; Nighttime, 6:00 p.m–6:00 a.m; urban medical regions, Sapporo, Kamikawa Chubu, and Minami Oshima medical regions; rural medical regions, regions outside the urban medical regions

* P < 0.05

### Factors associated with clinical outcomes

Considering possible collinearity, relationships of TIT, eDAD, medical region of onset, and type of transportation with the poor clinical outcome were examined using separate models of multiple logistic regression analysis (Table 4). Age ≥ 75 years, Killip class 3/4 on arrival, and onset in a rural medical region with inter-facility transfer were independently associated with CPC 3–5 (Table 4, model 1). In contrast, an association between poor outcome and TIT or eDAD was not detected (Table 4, models 2 and 3). No EMS call was negatively associated with poor outcomes. Additional file 1: Fig. S3 shows the frequency distributions of TIT in patients with good outcomes and those with poor outcomes in groups divided by medical regions of onset and types of transport to PCI facilities. The proportions of patients with short TIT were smaller in the groups of rural onset and inter-facility transfer, while there was no notable difference in the proportion of patients with poor outcomes between the groups.

**Table 4:** Factors associated with poor outcome (CPC 3–5)

	Model 1			Model 2			Model 3		
	OR	95% CI	P value	OR	95% CI	P value	OR	95% CI	P value
Age ≤ 64 years	1.00	[Ref.]		1.00	[Ref.]		1.00	[Ref.]	
65–74 years	2.55	0.99–6.54	0.05	2.46	0.97–6.21	0.06	2.51	0.99–6.34	0.05
≥ 75 years	6.17	2.65–14.36	< 0.01*	6.26	2.71–14.47	< 0.01*	5.72	2.48–13.21	< 0.01*
Women	0.66	0.31–1.44	0.30	0.68	0.32–1.44	0.31	0.69	0.33–1.48	0.34
Recurrent MI	0.83	0.32–2.15	0.71	0.77	0.30–1.95	0.58	0.77	0.30–1.97	0.59
No witness	0.82	0.44–1.55	0.55						
Onset in winter	1.09	0.58–2.06	0.79	1.01	0.55–1.86	0.98	1.05	0.56–1.97	0.88
Onset on weekends/holiday	0.86	0.43–1.71	0.66	0.96	0.49–1.87	0.89	0.98	0.50–1.94	0.96
Onset at night	1.27	0.68–2.39	0.45						
Killip class 3/4 on arrival	11.40	5.43–23.92	< 0.01*	11.76	5.65–24.45	< 0.01*	12.31	5.88–25.78	< 0.01*
No EMS call	0.38	0.17–0.85	0.02*						
Medical region and transport way									
Urban (direct admission)	1.00	[Ref.]							
Rural (direct admission)	1.30	0.61–2.79	0.50						
Urban (inter-facility transfer)	1.39	0.26–7.46	0.70						
Rural (inter-facility transfer)	2.79	1.17–6.66	0.02*						
Delays (per 1-h increase)									
TIT				0.93	0.83–1.06	0.28			
eDAD							0.95	0.82–1.09	0.47

Adjusted odds ratio (OR) and 95% confidence interval (CI) indicate the likelihood of poor outcome (CPC 3–5) from logistic regression analysis. OR > 1 indicates increased odds of poor outcome (CPC 3–5). Respective reference categories = age ≤ 64 years, male gender, first occurrence of MI, presence of a witness, onset in summer, onset on a weekday, onset in the daytime, Killip class 1/2 on arrival, and urban medical region (direct admission). CPC, cerebral performance category; MI, myocardial infarction; Daytime, 6:00 a.m–6:00 p.m; Nighttime, 6:00 p.m–6:00 a.m; EMS, emergency medical services; TIT, total ischemic time; eDAD, estimated delay-in-arrival-to-door

* P < 0.05

## Discussion

A total of 1341 STEMI patients in the 8-month registry were enrolled in this study. Although the number of cases in the registry is relatively small, the crude incidence of STEMI was calculated to be 37.4 (/100,000 persons/year), similar to previous Japanese registry data [20, 21]. In addition, a high percentage (86%) of PCI facilities in Hokkaido participated in the survey, indicating that there was probably minimal selection bias among the study subjects and facilities.

The median TIT in the present study cohort was 185 min, which is comparable to TIT data in high-income countries (2.0–4.0 h) [22–24]. Factors that were shown to be associated with prolonged TIT in previous studies include age, sex, type of symptoms, misinterpretation of the disease by patients or medical practitioners, absence of a witness, time of onset, use of EMS, social class, and distance from a hospital at the time of onset [15, 18, 24–28]. Older patients are more likely to have atypical or asymptomatic symptoms [27]. Sex differences were attributed to age, comorbidities, symptoms, social support, and insurance characteristics [25, 27]. Social and emotional factors can also contribute to delays in seeking care. For example, concern for social propriety can delay care-seeking because people do not want to trouble others. Additionally, patients may feel embarrassed when symptoms occur during off-hours and when they consider the possibility that their symptoms are not severe [28]. Consistent with earlier findings, advanced age, absence of a witness, nighttime onset, no EMS call, and symptom onset in a rural region (without direct transfer) were associated with prolonged TIT in the present study (Table 2).

As expected, ODT and TIT in the Direct ambulance-transport group were the shortest among the four groups with different transport pathways (Fig. 3). DBT in the inter-facility transport group tended to be shorter than that in the direct transport group. The reason why DBT in the inter-facility transfer group tended to be shorter than that in the direct transfer group is that the diagnosis of AMI had already been made at the previous medical facility, and the time for treatment and explanation was therefore reduced at the medical facility to which the patient was transferred. However, the shortening was not sufficient to compensate for the time delay caused by going through a non-PCI facility. These results are consistent with those of earlier studies [29, 30]. The proportion of ODT in TIT was much larger than that of DBT, confirming that prehospital delay is a significant cause of TIT prolongation.

The proportion of STEMI patients with ODT ≤ 120 min was 56% in the present registry (Fig. 4A). One of the major components of ODT is the patient’s decision time [25], and thus public education programs for improving the recognition of AMI and promoting the use of EMS have been conducted in some countries since the 1980s. However, such public interventions have not markedly changed the situation, and a recent study in Germany showed that TIT instead increased from 1994 to 2002 [26]. In the present study, we used pretransport time as an index of the patient’s decision time and it was found that pretransport time significantly differed between the Direct ambulance-transport group and the Inter-facility ambulance-transport group (median 40 vs. 63 min) (Fig. 3). Older age and nocturnal onset were associated with pretransport time prolongation (Table 2), which may be due to differences in symptoms and/or patient responsiveness to those symptoms [31]. These findings suggest that public and patient education should focus on the elderly and appropriate treatment-seeking behavior for STEMI symptoms at night. Interestingly, winter onset was associated with shorter pretransport time, despite the fact that AMI patients admitted in the cold season tend to have worse severity and prognosis [32]. This may be related to shorter pretransport time in winter, particularly in the interval from onset to EMS call (as shown in Additional file 1: Table S1).

The length of ODT for STEMI patients can be influenced not only by patient and hospital factors but also by factors related to patient transportation, such as geographical distance, number of routes from the onset location to a PCI facility, and availability of EMS. A study by Jena et al. showed that infrastructural factors could have a significant impact on outcomes of patients with myocardial infarction [33]. They showed that ambulance travel time was prolonged by 4.4 min and that adjusted 30-day mortality increased by 3.4% during big city marathons that involved widespread road closure and diversion of the EMS. In the present study, ODT was analyzed separately as a factor dependent on geographic infrastructure, min-PST, and as a factor dependent on other factors, eDAD, in order to identify appropriate and effective interventions. The percentages of patients in the short eDAD group who had onset in urban medical regions and did not across medical regions were higher than those in the long eDAD group (Additional file 1: Table S2). Emergency medical teams in Japan are organized by medical region, and Hokkaido has 21 medical regions (Additional file 1: Fig. S1A and B). Therefore, patients tend to be initially transported to a medical institution in the medical region to which they belong, even if there is a hospital nearby in a neighboring medical region and if the hospital is far away or is a non-PCI facility. The shorter eDAD in urban medical regions may be due to more PCI facilities and fewer inter-facility transfers, making it less likely for a second eDAD to occur. The factors related to eDAD prolongation included not only factors associated with pretransport time prolongation (i.e., older age and nighttime onset) but also no EMS call and inter-facility transfer, regardless of regional differences (Table 3). These findings suggest that it may be possible to shorten eDAD, even under the condition of the current geographic infrastructure, by increasing the proportion of direct EMS transport. On the other hand, there are certain areas in Hokkaido where min-PST is unacceptably long (Additional file 1: Fig. S2). In these areas, it was found that patient guidance and the efforts and cooperation of the EMS team could not effectively shorten ODT. This suggests that other treatment strategies or improving the geographic infrastructure may be necessary to reduce ODT in these areas.

The cumulative frequency distribution of ODT showed that ODT was more than 120 min in nearly half of the STEMI patients enrolled in the survey (Fig. 4A). This indicates that further interventions are necessary to shorten ODT in Hokkaido. However, if eDAD is eliminated, only "true" geographic infrastructure-dependent time remains, and ODT is projected to be less than 120 min in more than 90% of cases in Hokkaido (Fig. 4B). This projection suggests that eDAD, as well as the infrastructure for STEMI patient transport, is an important target to increase the number of STEMI patients who can receive timely PCI. In addition, the projection indicates that at least a few percent of patients cannot receive timely PCI. In such cases, fibrinolytic therapy [1–3, 34] (which is not common in Japan [23]) or air medical services to transport patients to PCI facilities [35] may be options.

Poor clinical outcome in the present study was predicted by age ≥ 75 years, Killip class 3/4 on arrival, and EMS call as in earlier studies [16, 17] (Table 4, Model 1). Onset in a rural region with inter-facility transfer was also associated with poor outcomes. Differences in medical resources and the extension of TIT may explain the association. Efforts to increase the percentage of direct transport and the previously mentioned fibrinolytic therapy or utilization of air medical services may provide solutions. On the other hand, the association between prolonged TIT and worsening clinical outcomes in STEMI patients has been inconsistent in observational studies [9, 36], and such an association was not found in the present study subjects (Table 4, Model 2). Possible explanations for this inconsistency include confounding and selection bias in study subjects [9], misalignment of onset of coronary occlusion and symptom onset, lack of consideration of parameters that influence the severity of myocardial ischemia such as presence or absence of pre-infarct angina [37], and differences in length of the follow-up period between the studies. Although the impact of short TIT on the outcome of STEMI remains controversial, an attempt to shorten TIT would be justified since it potentially increases the benefit of primary PCI without specific adverse effects.

### Study limitations

There are several limitations of the present study. First, this study was a non-randomized observational study; thus, causal relationships between the parameters and/or outcomes cannot be concluded. Second, there may have been some selection bias for enrolment because the present study included only patients with information for the onset of symptoms, reaching a PCI facility, and undergoing primary PCI. Third, we could not use cardiac function, heart failure, or cardiovascular death as clinical endpoints since they were not prespecified in data collection sheets in the Hokkaido Acute Coronary Care Survey. Fourth, data for medications prior to STEMI onset, comorbidities, and therapy afforded in non-PCI facilities were unavailable and could not be used to adjust the study subjects' clinical outcomes. Finally, consumer-available GIS software does not take into account the difference between standard ground transportation time and ambulance transportation time, and eDAD may therefore have been underestimated in this study.

## Conclusions

For STEMI patients enrolled in a prefectural level survey, it was possible to calculate an index of geographical infrastructure-independent prehospital time delay, eDAD, using a geographical information system. eDAD prolongation was predicted by no witness, onset at night, no EMS call, and inter-facility transfer in urban or rural medical regions. When eDAD was postulated to be zero in all cases, ODT was projected to be less than 120 min in > 90% of patients in the study prefecture. Thus, interventions to shorten eDAD by focusing on geographical infrastructure-independent factors for its prolongation may be an important strategy for shortening ODT. Moreover, the quality of STEMI patient transport in areas with different geographical conditions may be assessable based on the eDAD-ODT relationship.

## Supplementary Information

**Additional file 1: Fig. S1A**. Distribution of cardiology hospitals in Hokkaido in 2017. The numbers of cardiology hospitals were large in Sapporo, Minami Oshima, and Kamikawa Chubu medical regions. (109, 21, and 18, respectively). **Fig. S1 B**. Distribution of cardiologists (/100,000 persons) in Hokkaido in 2016. The numbers of cardiologists per 100,000 population were large in Sapporo, Minami Oshima, and Kamikawa Chubu. medical regions (16.0, 12.8 and 12.4, respectively). **Fig. S2**. Mapping of PCI facillities and min-PST data in Hokkaido. ArcGIS Online (ESRI, Inc., Charlotte, NC, USA) was used to determine min-PST, a time to the nearest PCI facility, and it was color-coded. PST, prehospital system time; PCI, percutaneous coronary intervention. **Fig. S3**. Frequency distributions of TIT in patients with good and poor outcomes in groups divided by medical regions of onset and types of transport. The figure shows the frequency distributions of TIT in patients with good outcomes and those with poor outcomes in groups divided by medical regions of onset and types of transport to PCI facilities. Numbers indicate time intervals as medians (interquartile range). CPC, cerebral performance category; TIT, total ischemic time; urban medical regions, Sapporo, Kamikawa Chubu, and Minami Oshima medical regions; rural medical regions, regions outside the urbanmedical regions. **Table S1**. Comparison of Seasons in pretransport time and its components. **Table S2**. Comparison of Two Groups by Median eDAD.

## Data Availability

The data that support the findings of this study are available from the Hokkaido Government, which is a public organization, but restrictions apply to the availability of these data, which were used under license for the current study and are therefore not publicly available. Data are however available from the authors upon reasonable request and with permission of the Hokkaido Government.

## Abbreviations

AMI: Acute myocardial infarction
CPC: Cerebral performance category
CPA: Cardiopulmonary arrest
DBT: Door-to-balloon time
DIDO: Door-in-to-door-out time
eDAD: Estimated delay-in-arrival-to-door
EMS: Emergency medical services
FMCTB: First-medical-contact-to-balloon time
GIS: Geographical information system
JCS: Japan Coma Scale
min-PST: Minimum prehospital system time
ODT: Onset-to-door time
OHCA: Out-of-hospital cardiac arrest
PCI: Percutaneous coronary intervention
PST: Prehospital system time
STEMI: ST-segment elevation myocardial infarction
TIT: Total ischemic time
