# Supplementary material for: Characterization of prehospital time delay in primary percutaneous coronary intervention for acute myocardial infarction: analysis of geographical infrastructure-dependent and -independent components
Source: Int J Health Geogr. 2023 Mar 30;22:7. doi: 10.1186/s12942-023-00328-5 (PMC10064653; doi:10.1186/s12942-023-00328-5)
Supplement: Supplementary file 1 — Additional file 1: Fig. S1A. Distribution of cardiology hospitals in Hokkaido in 2017. The numbers of cardiology hospitals were large in Sapporo, Minami Oshima, and Kamikawa Chubu medical regions. (109, 21, and 18, respectively). Fig. S1 B. Distribution of cardiologists (/100,000 persons) in Hokkaido in 2016. The numbers of cardiologists per 100,000 population were large in Sapporo, Minami Oshima, and Kamikawa Chubu. medical regions (16.0, 12.8 and 12.4, respectively). Fig. S2. Mapping of PCI facillities and min-PST data in Hokkaido. ArcGIS Online (ESRI, Inc., Charlotte, NC, USA) was used to determine min-PST, a time to the nearest PCI facility, and it was color-coded. PST, prehospital system time; PCI, percutaneous coronary intervention. Fig. S3. Frequency distributions of TIT in patients with good and poor outcomes in groups divided by medical regions of onset and types of transport. The figure shows the frequency distributions of TIT in patients with good outcomes and those with poor outcomes in groups divided by medical regions of onset and types of transport to PCI facilities. Numbers indicate time intervals as medians (interquartile range). CPC, cerebral performance category; TIT, total ischemic time; urban medical regions, Sapporo, Kamikawa Chubu, and Minami Oshima medical regions; rural medical regions, regions outside the urbanmedical regions. Table S1. Comparison of Seasons in pretransport time and its components. Table S2. Comparison of Two Groups by Median eDAD. [file 12942_2023_328_MOESM1_ESM.pdf]

**Additional file 1**

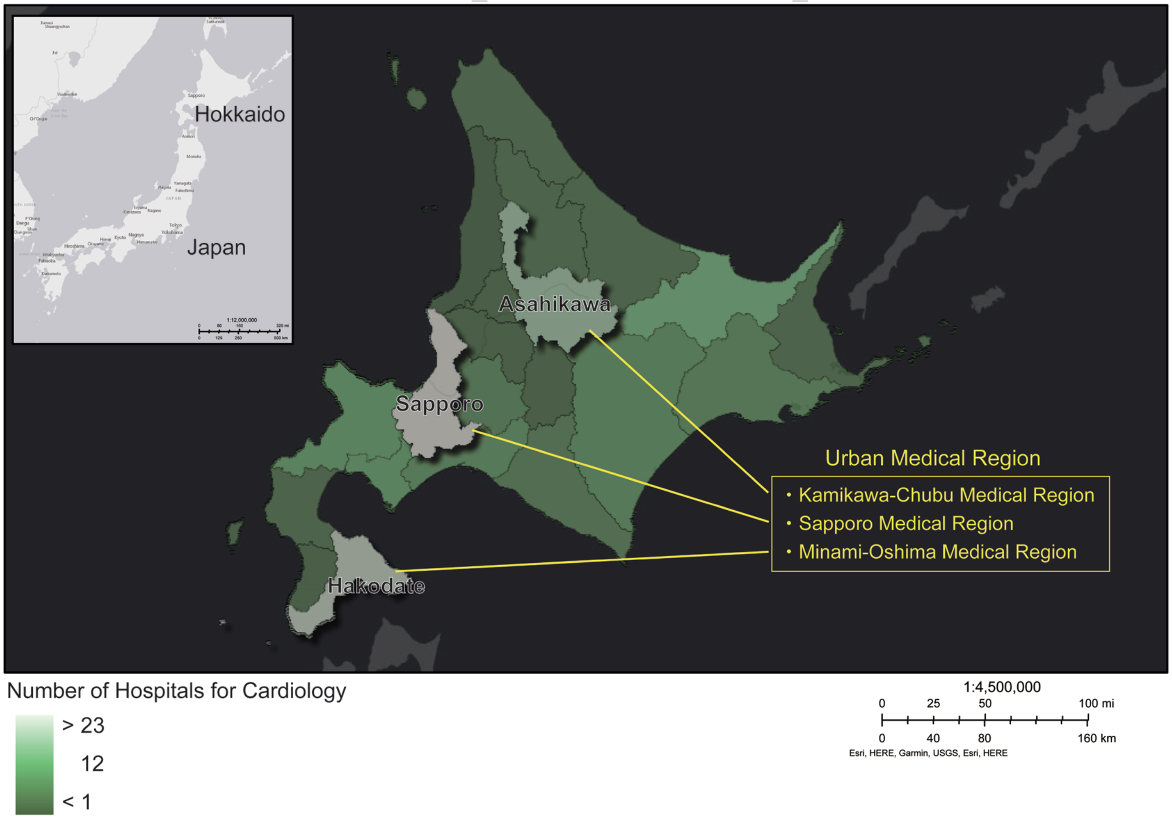


**Fig. S1A** Distribution of cardiology hospitals in Hokkaido in 2017. The numbers of cardiology hospitals were large in Sapporo, Minami Oshima, and Kamikawa Chubu medical regions (109, 21, and 18, respectively).

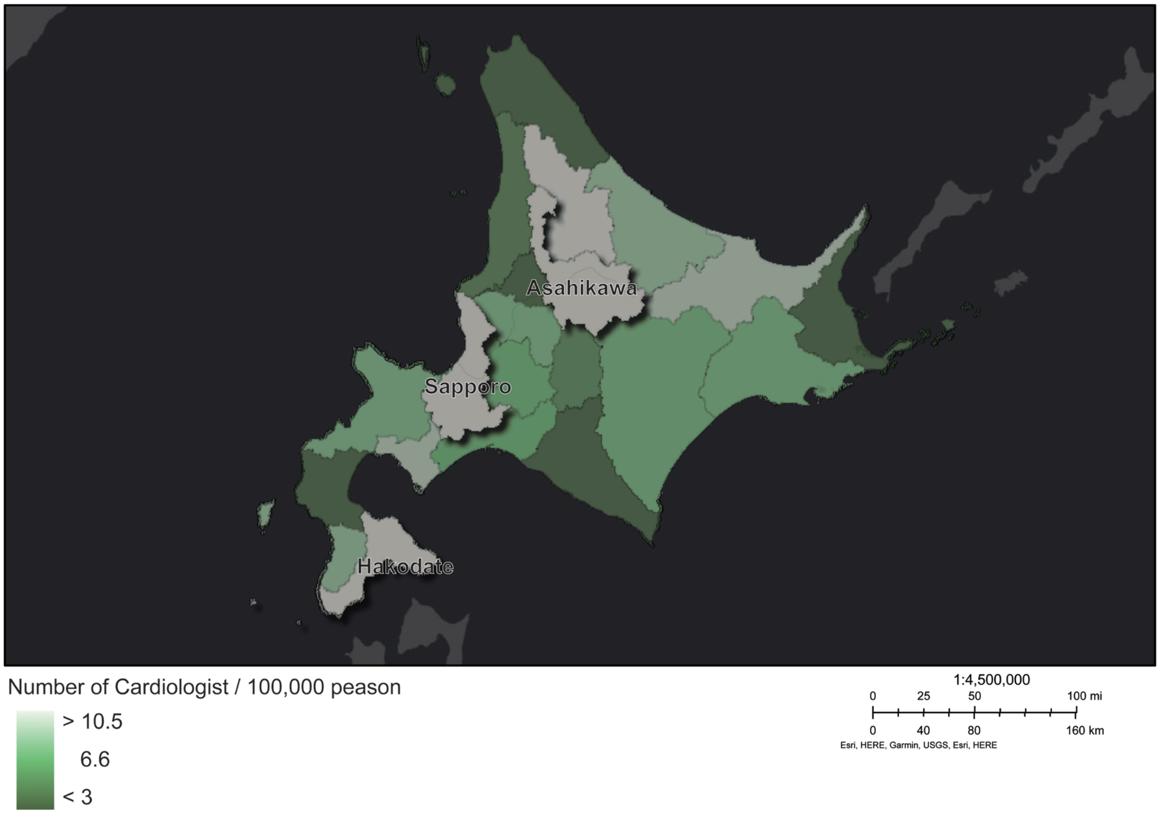


**Fig. S1B** Distribution of cardiologists (/100,000 persons) in Hokkaido in 2016. The numbers of cardiologists per 100,000 population were large in Sapporo, Minami Oshima, and Kamikawa Chubu medical regions (16.0, 12.8 and 12.4, respectively).

**Table S1** Comparison of seasons in pretransport time and its components

| | Summer (n = 192) | Winter (n = 213) | P value |
|---|---|---|---|
| Pretransport Time, min | 55 (25, 120) | 36 (19, 83) | < 0.01* |
| Onset to EMS call, min | 45 (17, 113) | 29 (10, 73) | < 0.01* |
| EMS call to FMC, min | 7 (5, 8) | 7 (6, 10) | 0.06 |

Data are expressed as median (interquartile range) for continuous variables. P values for the overall comparisons were calculated using the Wilcoxon rank-sum test. *EMS*, emergency medical services; *FMC*, first medical contact.

* P < 0.05

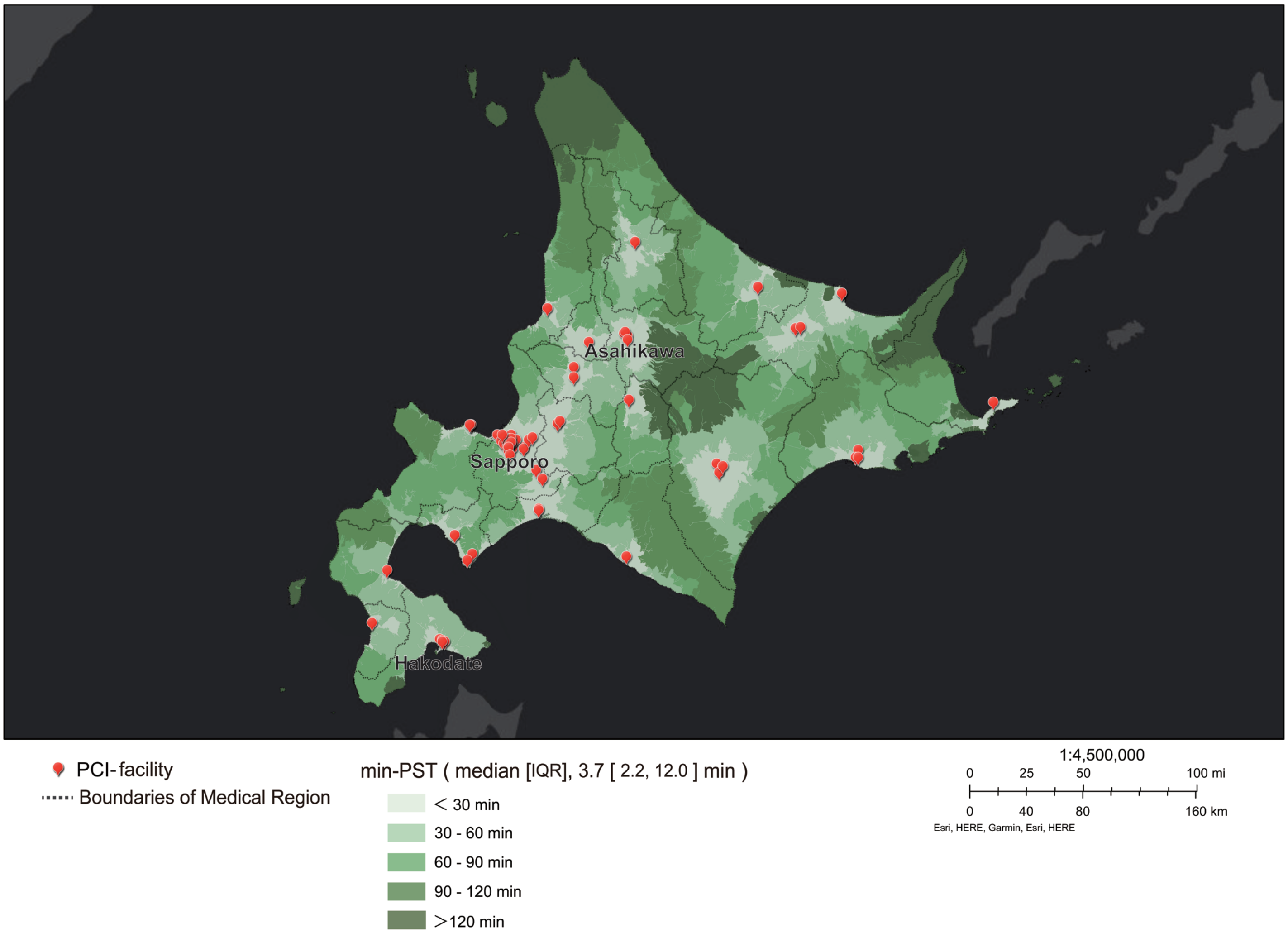


**Fig. S2** Mapping of PCI facilities and min-PST data in Hokkaido. ArcGIS Online (ESRI, Inc., Charlotte, NC, USA) was used to determine min-PST, a time to the nearest PCI facility, and it was color-coded. *PST*, prehospital system time; *PCI*, percutaneous coronary intervention.

**Table S2** Comparison of Two Groups by Median eDAD

| | short eDAD (n=297) | long eDAD (n=297) | P value |
|---|---|---|---|
| **Demographic characteristics and history** | | | |
| Age, years (mean ± SD) | 66.9 ± 12.4 | 68.8 ± 12.7 | 0.064 |
| Women, n (%) | 73 (24.6) | 65 (21.9) | 0.437 |
| **Presentation at PCI-facility** | | | |
| Killip class on arrival | | | |
| 1 / 2, n (%) | 200 (67.3) | 212 (71.4) | 0.487 |
| 3 / 4, n (%) | 44 (14.8) | 42 (14.1) | |
| Unknown, n (%) | 53 (17.9) | 43 (14.5) | |
| **Comparison of situations at symptom onset** | | | |
| Location at STEMI onset | | | |
| Urban medical regions, n (%) | 169 (56.9) | 140 (47.1) | 0.017* |
| Rural medical regions, n (%) | 128 (43.1) | 157 (52.9) | |
| Across medical regions | | | |
| Yes, n (%) | 31 (10.4) | 54 (18.2) | 0.007* |
| No, n (%) | 266 (89.6) | 243 (81.8) | |
| Season | | | |
| Summer, n (%) | 130 (43.8) | 146 (49.2) | 0.188 |
| Winter, n (%) | 167 (56.2) | 151 (50.8) | |
| Day of onset | | | |
| Weekday, n (%) | 204 (69.2) | 208 (70.0) | 0.816 |
| Weekend or Holiday, n (%) | 91 (30.9) | 89 (30.0) | |
| Time of onset | | | |
| Daytime, n (%) | 191 (64.3) | 168 (56.6) | 0.054 |
| Nighttime, n (%) | 106 (35.7) | 129 (43.4) | |
| **Transport path** | | | |
| EMS call | | | |
| Yes, n (%) | 246 (82.8) | 155 (52.2) | <0.001* |
| No, n (%) | 51 (17.2) | 142 (47.8) | |
| Use of air transportation, n (%) | 3 (7.9) | 3 (2.4) | 0.144 |
| **Time course** | | | |
| TIT, min (median [IQR]) | 124 (100, 155) | 285 (210, 410) | <0.001* |
| ODT, min (median [IQR]) | 56 (40, 76) | 203 (138, 307) | <0.001* |
| DBT, min (median [IQR]) | 59 (43, 88) | 65 (45, 92) | 0.366 |
| FMCTB, min (median [IQR]) | 88 (66, 117) | 110 (78, 164) | <0.001* |
| DIDO, min (median [IQR]) | 29 (12, 50) | 43 (13, 75) | 0.040* |
| mPST, min (median [IQR]) | 3 (2, 10) | 6 (2, 14) | 0.001* |
| eDAD, min (median [IQR]) | 49 (35, 64) | 179 (124, 290) | <0.001* |
| **Outcome** | | | |
| Death (CPC 5), n (%) | 20 (6.7) | 18 (6.1) | 0.737 |
| Poor (CPC 3–5), n (%) | 26 (8.8) | 28 (9.4) | 0.775 |
| Good (CPC 1–2), n (%) | 271 (91.3) | 269 (90.6) | |

We simulated min-PST (travel time from each onset point to the nearest PCI facility) using ArcGIS Pro (ESRI, Inc., Charlotte, NC, USA). eDAD (estimated delay-in-arrival-to-door; ODT minus min-PST) means the amount of room for improvement. Variables are expressed as numbers (%), means ± standard deviation (SD), or medians (IQR; interquartile ranges). ODT, onset to door time; CPA, cardiopulmonary arrest; urban medical regions, Sapporo, Kamikawa Chubu, and Minami Oshima medical regions; rural medical regions, regions outside the urban medical regions; Daytime, 6:00 a.m–6:00 p.m; Nighttime, 6:00 p.m–6:00 a.m; *EMS*, emergency medical system; *TIT*, total ischemic time; *ODT*, onset-to-door-time; *min-PST*, minimum prehospital system time, *DBT*, door-to-balloon-time; *FMCTB*, first-medical-contact-to-balloon-time; *PTT*, pretransport time, *DIDO*, door-in-to-door-out-time; *CPC*, cerebral performance category.

* $P < 0.05$

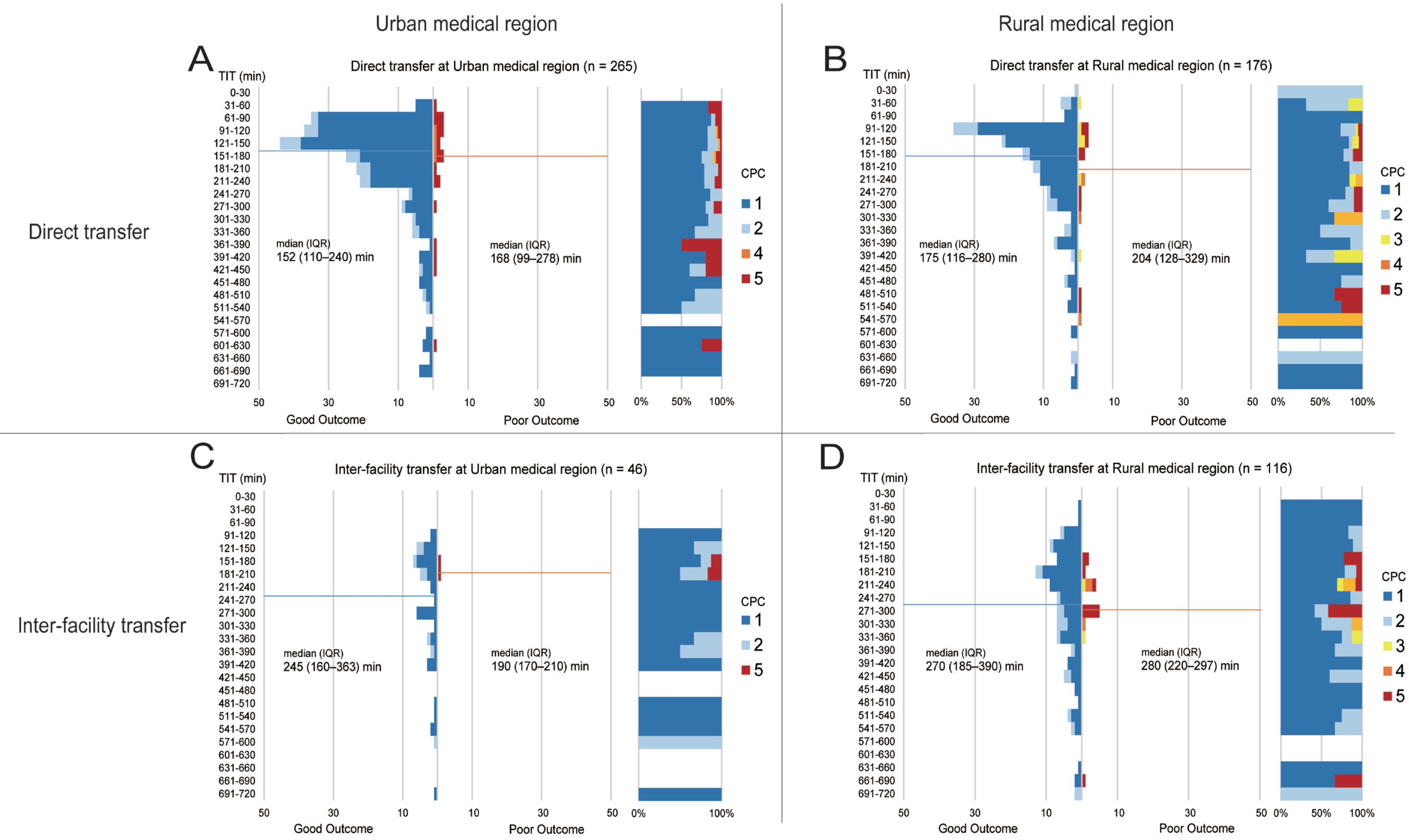


**Fig. S3** Frequency distributions of TIT in patients with good and poor outcomes in groups divided by medical regions of onset and types of transport. The figure shows the frequency distributions of TIT in patients with good outcomes and those with poor outcomes in groups divided by medical regions of onset and types of transport to PCI facilities. Numbers indicate time intervals as medians (interquartile range). *CPC*, cerebral performance category; *TIT*, total ischemic time; urban medical regions, Sapporo, Kamikawa Chubu, and Minami Oshima medical regions; rural medical regions, regions outside the urban medical regions.
